# Safety and Efficacy of Specially Designed Texture-Modified Foods for Patients with Dysphagia Due to Brain Disorders: A Prospective Study

**DOI:** 10.3390/healthcare9060728

**Published:** 2021-06-13

**Authors:** Soyoung Kwak, Yoo Jin Choo, Kyu Tae Choi, Min Cheol Chang

**Affiliations:** Department of Physical Medicine & Rehabilitation, College of Medicine, Yeungnam University, Daegu 42415, Korea; sk315@ynu.ac.kr (S.K.); cyj361@hanmail.net (Y.J.C.); choi3190@ynu.ac.kr (K.T.C.)

**Keywords:** dysphagia, texture-modified foods, dysphagia diet, brain disorders, texture analysis

## Abstract

Providing texture-modified food for patients with dysphagia is a cornerstone of dysphagia treatment. This study aimed to evaluate the safety and efficacy of a specially designed texture-modified food that can be easily swallowed while maintaining the unique taste by adjusting hardness and adhesiveness in patients with brain disorders using a videofluoroscopic swallowing study. We included 101 patients with oropharyngeal dysphagia due to brain disorders who were referred to the rehabilitation department. To evaluate the safety and efficacy of a specially designed texture-modified food, rice gruel was compared with a regular instant rice porridge, and bulgogi mousse was compared with ground bulgogi, which normally serves as a texture-modified diet for patients with dysphagia in our hospital during the videofluoroscopic swallowing study. The Penetration–Aspiration Scale score, oropharyngeal transit time, number of swallows required to maximally eliminate food materials from the oropharyngeal space, and vallecular and pyriform sinus residue after swallowing scale score were compared. Rice gruel required a shorter oropharyngeal transit time and fewer number of swallowing per the given amount of food than regular instant rice porridge; however, no statistical difference was found in the vallecular and pyriform sinus residue after swallowing scale scores and the Penetration–Aspiration Scale scores. Bulgogi mousse required more swallowing and had lower Penetration–Aspiration Scale scores than ground bulgogi; however, no significant difference was found in the oropharyngeal transit time and the vallecular and pyriform sinus residue after swallowing scale scores. The study foods were safe and efficacious compared to control foods usually provided for patients with dysphagia from various brain disorders.

## 1. Introduction

Texture is one of the four principal quality factors in food, along with its appearance, flavor and nutrition [1]. It is not a single property; it is a group of physical properties derived from the structure of the food. Adhesiveness, cohesiveness, firmness, fracturability, hardness, springiness, viscosity and yield stress have been suggested to be the most significant components of texture in dysphagia diet and management [2]. Previous studies using kinematic analysis of dysphagia elucidated that bolus transit time and velocity are highly dependent on the patient’s medical conditions and food texture [3,4,5,6,7].

Providing texture-modified food for patients with dysphagia has been accepted as a cornerstone of dysphagia treatment [2,8,9]. However, previous studies have reported poor adherence to texture-modified diets due to limited choice, unsatisfactory taste, lack of experience and knowledge for preparing texture-modified diet, higher cost and longer time to prepare the foods as they require additional processing such as blending, grinding or chopping [10,11,12]. Poor adherence to texture-modified diet may lead to decreased oral intake, dehydration, increased risk of chest infection and malnutrition in patients with dysphagia [13,14,15]. In addition, no specific food texture has been demonstrated to have clear, measurable positive impact on the swallowing pattern [16,17]. Even though there are several clinical guidelines for dysphagia diet, most adopt viscosity category boundaries, which are based on consensus rather than evidence [16]. Therefore, it is important to develop texture-modified foods that can address the aforementioned issues.

In this study, we aimed to evaluate the safety and efficacy of specially designed texture-modified foods (rice gruel and bulgogi mousse) for patients with dysphagia that can be easily swallowed while maintaining the unique taste of foods by adjusting their hardness and adhesiveness using videofluoroscopic swallowing study (VFSS). This study aimed to compare the airway safety measured by the Penetration–Aspiration Scale (PAS) scores of the study and control foods and compare the efficacy of the study and control foods using the oropharyngeal transit time, number of swallows required to eliminate food materials from the oropharyngeal space, and the amount of residue in the pharynx after swallowing. 

## 2. Materials and Methods

### 2.1. Study Design

**Figure 1:**
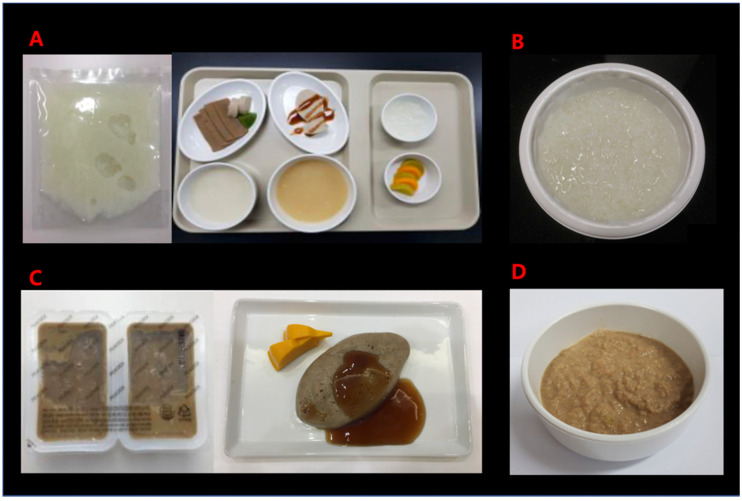
Study and control foods. (**A**) rice gruel; (**B**) rice porridge; (**C**) bulgogi mousse; and (**D**) ground bulgogi.

The study foods were rice gruel and bulgogi mousse (Shinsegae Food Inc., Seoul, Korea). To evaluate the safety and efficacy of the study foods, rice gruel was compared with a regular instant rice porridge widely available in the market and bulgogi mousse was compared with ground bulgogi, which normally serves as a texture-modified diet for patients with dysphagia in our hospital (Figure 1). Since it was impractical to compare the VFSS findings of the study and control food in different patients controlling for potential confounding factors, the study and control foods were tested in the same patient. Since we had two pairs of study and control foods, the patients were divided into two different groups—the rice gruel group and the bulgogi mousse group- to avoid excessive radiation exposure. 

### 2.2. Participants

The inclusion criteria were as follows: (1) patients who were referred to the rehabilitation department for VFSS; (2) patients who were diagnosed with stroke, traumatic brain injury, Parkinson’s disease, Alzheimer’s disease, brain tumor or hypoxic-ischemic encephalopathy; (3) patients who could sit and maintain upright posture during VFSS; (4) patients with alert mentality and sufficient cognitive function and could follow the directions during VFSS; and (5) patients confirmed to have oropharyngeal dysphagia in the VFSS. The exclusion criteria were as follows: (1) patients aged <20 years, (2) patients with a history of tracheostomy, (3) patients diagnosed with other diseases that can cause dysphagia such as neuromuscular diseases (including motor neuron disease and myopathies) or head and neck tumors, and (4) patients who were not able to complete VFSS without termination due to aspiration episodes more than once. Of the 380 patients who were referred to the rehabilitation department for VFSS during the study period, a total of 101 patients were enrolled in our study. Consequently, 50 patients were assigned to the rice gruel group and 51 patients to the bulgogi mousse group.

### 2.3. Videofluoroscopic Swallowing Study

VFSS was performed using an X-ray flat panel detector system (FPD, Zexira^®^, Toshiba, Tokyo, Japan). The patients were seated upright in the lateral viewing plane. The fluoroscopic images were digitally recorded at 30 frames per second using a scan converter. The VFSS study protocol for the rice gruel group included the following: (1) 3 mL of thin liquid (Bonorex 300 injection), (2) 5 mL of rice gruel, (3) 5 mL of rice porridge, (4) 5 mL of diced banana and (5) 10 mL of thin liquid (Bonorex 300 injection). The VFSS study protocol for the bulgogi mousse group included the following: (1) 3 mL of thin liquid, (2) 5 mL of ground bulgogi, (3) 5 mL of bulgogi mousse, (4) 5 mL of diced banana and (5) 10 mL of thin liquid. For patient safety, VFSS was terminated immediately after a second aspiration event, irrespective of the study stage. In this case, ten grams of each of the study and control food was mixed with 2 mL of Bonorex 300 injection (iohexol 647 mg/mL), and the temperature of all foods (except liquid Bonorex) was maintained at 40 °C–45 °C using a vacuum container during VFSS.

### 2.4. Outcome Measures

Airway safety was evaluated using PAS [18]. The efficacy of texture-modified foods was evaluated using oropharyngeal transit time (seconds), the number of swallows required to eliminate the food materials from the oropharyngeal space, and the amount of residue in the vallecular and pyriform sinuses after multiple swallows, using the vallecular and pyriform sinus residue after swallowing scale (grade 0: no residue and grades 1, 2 and 3: <10%, from 10% to 50%, and >50% of the width of vallecular or pyriform sinuses on the VFSS image, respectively) [19]. When the vallecular or pyriform sinus residue remained despite multiple swallows, the number of swallowing was determined as the smallest number that resulted in maximal clearance of the food materials. Two experienced raters independently provided was all the objective ratings. Discrepancies were resolved by consensus. 

### 2.5. Texture Analysis of the Study and Control Foods

The hardness and adhesiveness of rice gruel, rice porridge and bulgogi mousses were measured by compression tests using a texture analyzer. Texture analysis of ground bulgogi was not performed because the preparation of ground bulgogi under the same conditions as in our hospital was not possible. The hardness and adhesiveness of the aforementioned foods were obtained by mixing the foods with Bonorex 300 injection, as described above. The diameter of the probe cylinder was 2.0 cm, crosshead speed was 10 mm/s and load cell was 50 N. In addition, the Universal Design Foods (UDF) classification developed by the Japan Care Food Conference (Table 1) [20] was attached to the results. The temperature of the study and control foods was set at 20 °C–25 °C (at room temperature) and at 40 °C–50 °C (the temperature at which the foods are normally served in a real-world setting [e.g., in hospitals or at home]) to evaluate whether there were texture differences between the two temperature ranges, except for rice porridge mixed with Bonorex at a temperature of 40 °C–50 °C due to technical issues and the discontinuation of production of the control food during the study period. Texture analyses were performed three times for each food, and the mean values for each food were used to determine the UDF stage. Statistical analysis was not performed to determine the changes in the texture of the food after being mixed with Bonorex because the sample size was judged to be insufficient. All texture analyses were performed at the Food Processing Laboratory of Sejong University.

### 2.6. Standard Protocol Approvals, Registrations and Patient Consents

This study was conducted in accordance with the Declaration of Helsinki and was reviewed and approved by the Institutional Review Board of the University Hospital. Written informed consent was obtained from all participants.

### 2.7. Statistical Analysis

Data were analyzed using the Statistical Package for Social Sciences version 20.0 (IBM Corp., Armonk, NY, USA). Demographic data of the study participants were compared using the Mann-Whitney U test for age, Chi-squared test for gender and disease duration, and Fisher’s exact test for diagnosis. Among the outcome measures, the oropharyngeal transit time was compared using the paired *t*-test and the rest were compared using the Wilcoxon signed-rank test. All tests were two-tailed and uncorrected for multiple comparisons. Statistical significance was set at *p* < 0.05.

## 3. Results

A total of 101 patients were enrolled in this study (50 and 51 in the rice gruel and bulgogi mousse groups, respectively; Table 2). The mean patient age was 72 years in the rice gruel group and 72.73 years in the bulgogi mousse group. Furthermore, 25 of 50 patients in the rice gruel group and 23 of 51 patients in the bulgogi mousse group had a disease duration of ≥6 months. No significant difference was found in the demographic data of the participants in the rice gruel and bulgogi mousse groups.

According to the texture analysis of the study and control foods, rice gruel and rice porridge were classified as UDF stage 4 (food can be swallowed without chewing) and bulgogi mousse was classified as UDF stage 3 (food can be smashed with the tongue at temperatures of 20 °C–25 °C and 40 °C–50 °C; Table 3). The UDF stage of each food was maintained on mixing it with Bonorex 300, which was used as a contrast medium. 

After mixing with Bonorex 300, all the foods tested showed increased hardness at a temperature of 20 °C–25 °C; however, rice gruel showed decreased hardness and bulgogi mousse showed increased hardness at a temperature of 40 °C–50 °C. In terms of adhesiveness, all the foods tested showed decreased values after mixing with a contrast medium at all temperatures. 

### 3.1. Rice Gruel versus Rice Porridge

The oropharyngeal transit time of rice gruel was shorter than that of rice porridge (mean difference: 21.20 s, 95% confidence interval [CI], 14.97–27.43 s, *p* < 0.001; Table 4). In addition, rice gruel required a fewer swallows per given amount of food than rice porridge (*p* < 0.001). However, there was no statistically significant difference between the two foods regarding the vallecular and pyriform sinus residue after swallowing scale scores (*p* = 0.132 and *p* = 0.054, respectively) and PAS scores (*p* = 0.317).

### 3.2. Bulgogi Mousse versus Ground Bulgogi

There was no statistically significant difference between bulgogi mousse and ground bulgogi in terms of oropharyngeal transit time (*p* = 0.486; Table 4). However, bulgogi mousse required a greater number of swallows per given amount of food than ground bulgogi (*p* = 0.007). There was no significant difference in the vallecular and pyriform sinus residue after swallowing scale scores (*p* = 1.000 and *p* = 0.819, respectively) between bulgogi mousse and ground bulgogi. Lastly, the PAS score for bulgogi mousse was lower than that for ground bulgogi (*p* = 0.038).

## 4. Discussion

In this study, we aimed to evaluate the safety and efficacy of specially designed texture-modified foods in patients with brain disorders using VFSS. In summary, rice gruel required a shorter oropharyngeal transit time and fewer number of swallows per a given amount of food than rice porridge; however, there was no statistically significant difference between the two foods in terms of vallecular and pyriform sinus residue after swallowing scale and PAS scores. Hence, rice gruel has superior efficacy and comparable safety compared to rice porridge. Foods with lower hardness and adhesiveness are easier to swallow in healthy subjects [21]. Both rice gruel and rice porridge were classified as UDF stage 4. However, as rice gruel has lower hardness and adhesiveness than rice porridge in texture analysis, it might be expected that rice gruel would be associated with fewer residues after swallowing, a shorter oropharyngeal transit time and fewer swallows per a given amount of food than rice porridge, and the PAS scores for rice gruel and rice porridge would be similar because both foods are classified into the same UDF stage. The study results were similar to this; however, there was no significant difference in the amount of vallecular and pyriform sinus residues between the two foods. This might be ascribed to the fact that after mixing with contrast medium, the adhesiveness of both foods and the difference in adhesiveness between the two foods decreased, as shown in Table 3. However, limited information is available regarding the extent to which adhesiveness may result in pharyngeal residues. Further studies are needed to clarify the association between adhesiveness and pharyngeal residues after swallowing.

In contrast, bulgogi mousse required a greater number of swallows than ground bulgogi; however, no significant difference was found between the two foods in terms of the oropharyngeal transit time and the vallecular and pyriform sinus residue after swallowing scale scores. Further, the PAS scores for bulgogi mousse were lower than those for ground bulgogi. Dissimilar to rice gruel and rice porridge, texture analysis was not performed for ground bulgogi, which is a control food for bulgogi mousse. However, the UDF classification of ground bulgogi was stage 4 (food can be swallowed without chewing), while that of bulgogi mousse was stage 3 (food can be smashed with the tongue). We believe that the difference in the texture of bulgogi mousse and ground bulgogi is responsible for the higher number of swallows for bulgogi mousse than for ground bulgogi. However, the overall oropharyngeal transit time was not statistically different between the two foods; hence, bulgogi mousse and ground bulgogi can be considered to have comparable efficacy. The superior safety of bulgogi mousse, represented as a significantly lower PAS score, could result from differences in the texture of the ground bulgogi and bulgogi mousse. It appears that the viscosity of ground bulgogi was lower than that of bulgogi mousse, which is compatible with the results of previous studies that reported that boluses with lower viscosity are more difficult to swallow safely than boluses with higher viscosity [22,23]. However, objective texture analysis for ground bulgogi was not performed in this study; thus, further studies are needed to elucidate its association. It should also be noted that despite the statistical significance, the mean difference in PAS scores might not be significant in the clinical setting. 

Several previous studies have identified the beneficial effect of high shear viscosity in reducing the risk of aspiration during swallowing. It is assumed that high-viscosity boluses are transported more slowly than low-viscosity boluses, thus allowing more time for the oropharyngeal mechanism to secure the airways until the bolus enters the esophagus [9]. Therefore, the nutritional management of patients with dysphagia is based on increasing the viscosity to a certain range that is considered safe for swallowing. However, very thick foods are poorly accepted by patients, and high-viscosity foods may result in an increase in residues after swallowing, reduction of palatability and increased risk of dehydration [24,25]. Therefore, it is important for clinicians to understand the physical properties of foods and their relation to dysphagia and be more specific in prescribing texture-modified diets for patients with dysphagia. 

However, the assessment and application of texture modification is subjective in clinical settings; the preparation as well as the rheological evaluation and administration of thickened foods to patients are universally subjective and a wide range of viscous properties are recommended for the same level of dysphagia [26,27]. In addition, despite the established knowledge on the close connection between dysphagia and rheological properties of food bolus, knowledge has not been incorporated into the national guidelines for the dietary management of dysphagia, such as the National Dysphagia Diet Task Force (2002) of the American Dietetic Association [28] or the British Dietetic Association (2009) in the National Description for Texture Modification in Adults [29]. However, attempts have been made to establish an international terminology for texture-modified foods used in dysphagia management [8,30,31].

The results of this study are meaningful because to the best of our knowledge, this is the first study to evaluate the safety and efficacy of specially designed dysphagia food in patients with brain disorders using VFSS. Previous studies have focused mainly on the nutritional state of patients with dysphagia [12,31,32,33], but limited information is available regarding the safety and efficacy of swallowing foods with different physical properties other than viscosity using VFSS. 

### Study Limitations

First, the lack of randomization of the sequence of study and control foods is a major limitation of this study. Due to the limited staffing, blinding for the sequence was not possible, and this could lead to bias. Second, texture analyses of ground bulgogi and rice porridge mixed with Bonorex at a temperature of 40 °C–50 °C were not performed. In addition, the ease of preparation (compared to conventional texture-modified foods prepared at home), adherence to the prescribed diet, patient satisfaction and long-term outcomes such as nutritional state or frequency of chest infection, were not included in the current study.

## 5. Conclusions

In conclusion, the specially designed texture-modified foods—rice gruel and bulgogi mousse—were found to be safe and efficacious for patients with dysphagia and various brain disorders. Therefore, practical application of these foods is warranted. 

## Figures and Tables

**Table 1:** The Universal Design Foods guidelines for elderly people.

	Stage 1	Stage 2	Stage 3	Stage 4
Classifications	Able to chew easily	Able to smash with gums	Able to smash with tongue	Able to swallow without chewing
Standards of chewing	Contains hard and big ingredients, a little hard to swallow	Contains hard and big ingredients not easy to swallow	Contains soft and small ingredients, easy to swallow	Hard to swallow if contains solid food
Standards of swallowing	Able to swallow commonly	Depends on ingredients, hard to swallow	Have experience hard to swallow water or liquid ingredients	Hard to swallow water or liquid ingredients
Hardness (N/m^2^)	5 × 10^5^	5 × 10^4^	2 × 10^4^	5 × 10^3^

Reproduced from [20] via license: CC BY-NC 4.0. No changes were made to this table.

**Table 2:** Demographic data of the study participants.

	Rice Gruel vs. Rice Porridge	Bulgogi Mousse vs. Ground Bulgogi	*p*-Value
Age (years) (mean ± SD)	72.00 ± 15.17	72.73 ± 12.05	0.736 ^a^
Male: Female (n)	23:27	26:25	0.482 ^b^
Diagnosis (n)			0.350 ^c^
Ischemic stroke	23	27	
Hemorrhagic stroke	13	6	
Alzheimer’s disease	6	12	
Parkinson’s disease	4	3	
Brain tumor	2	1	
Hypoxic ischemic brain injury	1	0	
Traumatic brain injury	1	2	
Disease duration (n)			0.622 ^b^
<6 months	25	28	
≥6 months	25	23	

Notes: Values are presented as numbers or mean ± standard deviation. ^a^ *p*-value was calculated by the Student’s *t*-test. ^b^ *p*-value was calculated by the Chi-squared test. ^c^ *p*-value was calculated by the Fisher’s exact test. SD, standard deviation.

**Table 3:** Results of text analysis of the study and control food.

Temperature		Rice Gruel	Bulgogi Mousse	Rice Porridge	Rice Gruel + Bonorex	Bulgogi Mousse + Bonorex	Rice Porridge + Bonorex
40~50 °C	Hardness (N/m^2^)	569 ± 76	4479 ± 359	1824 ± 240	483 ± 89	5603 ± 1882	-
	Adhesiveness (N·mm)	1.13 ± 0.07	4.01 ± 0.13	3.86 ± 0.54	0.33 ± 0.02	2.35 ± 0.72	-
	UDF stage	4	3	4	4	3	-
	Calories (kcal/100 g)	64.72	72.57	51.85	-	-	-
20~25 °C	Hardness (N/m^2^)	1049 ± 229	17,077 ± 648	1681 ± 41	1386 ± 109	18,871 ± 367	1894 ± 157
	Adhesiveness (N·mm)	1.23 ± 0.14	4.90 ± 0.28	4.60 ± 0.59	0.28 ± 0.04	4.00 ± 0.31	2.27 ± 0.47
	UDF stage	4	3	4	4	3	4

Notes: Values are presented as numbers or mean ± standard deviation.

**Table 4:** Comparison of outcome measure.

	Rice Gruel ^a^	Rice Porridge ^b^	*p*-Value	Bulgogi Mousse ^a^	Ground Bulgogi ^b^	*p*-Value
Oropharyngeal transit time (seconds)	39.88 ± 18.40	61.08 ± 28.59	<0.001 ^c^*	40.29 ± 28.03	37.51 ± 19.94	0.486 ^c^
Number of swallows required to maximally eliminate the food materials from the oropharyngeal space	4 (3;6)	5 (4;7)	<0.001 ^d^*	3 (2;4)	2 (2;3)	0.007 ^d^*
The vallecular residue after swallowing scale score	1 (1;1)	1 (1;1)	0.132 ^d^	1 (1;2)	1 (1;2)	1.000 ^d^
The pyriform sinus residue after swallowing scale score	1 (0;1)	1 (0;1)	0.054 ^d^	1 (0;1)	1 (0;1)	0.819 ^d^
The Penetration-Aspiration Scale score	1 (1;2)	1 (1;2)	0.317 ^d^	1 (1;1)	1 (1;2)	0.038 ^d^*

Notes: Values are presented as the mean for oropharyngeal transit time and median (Q1;Q3) for the rest. ^a^ Specially designed texture-modified foods for dysphagic people. ^b^ Control foods usually provided for dysphagic patients. ^c^ *p*-value was calculated by the Paired *t*-test. ^d^ *p*-value was calculated by the Mann-Whitney U test. * *p* < 0.05.

## Data Availability

The data presented in this study are available on request from the corresponding author.

## References

[1] Bourne M. (2002). Food Texture and Viscosity.

[2] Murry T., Carrau R.L., Chan K. (2006). Clinical Management of Swallowing Disorders.

[3] Takasaki K., Umeki H., Enatsu K., Tanaka F., Sakihama N., Kumagami H., Takahashi H. (2008). Investigation of pharyngeal swallowing function using high-resolution manometry. Laryngoscope.

[4] Bredenoord A.J., Smout A.J. (2008). High-resolution manometry. Dig. Liver Dis..

[5] Omari T.I., Rommel N., Szczesniak M.M., Fuentealba S., Dinning P.G., Davidson G.P., Cook I.J. (2006). Assessment of intraluminal impedance for the detection of pharyngeal bolus flow during swallowing in healthy adults. Am. J. Physiol. Gastrointest. Liver Physiol..

[6] Hasegawa A., Otoguro A., Kumagai H., Nakazawa F. (2005). Velocity of Swallowed Gel Food in the Pharynx by Ultrasonic Method. Nippon Shokuhin Kagaku Kogaku Kaishi.

[7] Lee S.H., Oh B.M., Chun S.M., Lee J.C., Min Y., Bang S.H., Kim H.C., Han T.R. (2013). The accuracy of the swallowing kinematic analysis at various movement velocities of the hyoid and epiglottis. Ann. Rehabil. Med..

[8] Hanson B. (2016). A review of diet standardization and bolus rheology in the management of dysphagia. Curr. Opin. Otolaryngol. Head Neck Surg..

[9] Ekberg O. (2019). Dysphagia: Diagnosis and Treatment.

[10] Flynn E., Smith C.H., Walsh C.D., Walshe M. (2018). Modifying the consistency of food and fluids for swallowing difficulties in dementia. Cochrane Database Syst. Rev..

[11] O’Keeffe S.T. (2018). Use of modified diets to prevent aspiration in oropharyngeal dysphagia: Is current practice justified?. BMC Geriatr..

[12] Painter V., Le Couteur D.G., Waite L.M. (2017). Texture-modified food and fluids in dementia and residential aged care facilities. Clin. Interv. Aging.

[13] Vucea V., Keller H.H., Morrison J.M., Duizer L.M., Duncan A.M., Carrier N., Lengyel C.O., Slaughter S.E., Steele C.M. (2018). Modified Texture Food Use is Associated with Malnutrition in Long Term Care: An Analysis of Making the Most of Mealtimes (M3) Project. J. Nutr. Health Aging.

[14] Cichero J.A.Y. (2018). Age-Related Changes to Eating and Swallowing Impact Frailty: Aspiration, Choking Risk, Modified Food Texture and Autonomy of Choice. Geriatrics.

[15] Shimizu A., Maeda K., Tanaka K., Ogawa M., Kayashita J. (2018). Texture-modified diets are associated with decreased muscle mass in older adults admitted to a rehabilitation ward. Geriat.r Gerontol. Int..

[16] Frazier J., Chestnut A.H., Jackson A., Barbon C.E., Steele C.M., Pickler L. (2016). Understanding the Viscosity of Liquids used in Infant Dysphagia Management. Dysphagia.

[17] Steele C.M., Alsanei W.A., Ayanikalath S., Barbon C.E., Chen J., Cichero J.A., Coutts K., Dantas R.O., Duivestein J., Giosa L. (2015). The influence of food texture and liquid consistency modification on swallowing physiology and function: A systematic review. Dysphagia.

[18] Rosenbek J.C., Robbins J.A., Roecker E.B., Coyle J.L., Wood J.L. (1996). A penetration-aspiration scale. Dysphagia.

[19] Han T.R., Paik N.J., Park J.W. (2001). Quantifying swallowing function after stroke: A functional dysphagia scale based on videofluoroscopic studies. Arch. Phys. Med. Rehabil..

[20] Kang A., Kim D., Kang S., Seo K., Park H., Park K. (2016). EMG Activity of Masseter Muscles in the Elderly According to Rheological Properties of Solid Food. Ann. Rehabil. Med..

[21] Tokifuji A., Matsushima Y., Hachisuka K., Yoshioka K. (2013). Texture, sensory and swallowing characteristics of high-pressure-heat-treated pork meat gel as a dysphagia diet. Meat Sci..

[22] Zargaraan A., Rastmanesh R., Fadavi G., Zayeri F., Mohammadifar M.A. (2013). Rheological aspects of dysphagia-oriented food products: A mini review. Food Sci. Hum. Wellness.

[23] Nyström M. (2015). Extensional Rheometry through Hyperbolic Contraction.

[24] Steele C.M., Molfenter S.M., Péladeau-Pigeon M., Stokely S. (2013). Challenges in preparing contrast media for videofluoroscopy. Dysphagia.

[25] Newman R., Vilardell N., Clavé P., Speyer R. (2016). Effect of Bolus Viscosity on the Safety and Efficacy of Swallowing and the Kinematics of the Swallow Response in Patients with Oropharyngeal Dysphagia: White Paper by the European Society for Swallowing Disorders (ESSD). Dysphagia.

[26] Steele C.M., Cichero J.A. (2008). A question of rheological control. Dysphagia.

[27] Steele C.M., Van Lieshout P.H., Goff H.D. (2003). The rheology of liquids: A comparison of clinicians’ subjective impressions and objective measurement. Dysphagia.

[28] National Dysphagia Diet Task F., American Dietetic A. (2002). National Dysphagia Diet: Standardization for Optimal Care.

[29] The British Dietetic Association and the Royal College of Speech and Language Therapist (2002). National Descriptors for Texture Modification in Adults.

[30] Cichero J.A., Steele C., Duivestein J., Clavé P., Chen J., Kayashita J., Dantas R., Lecko C., Speyer R., Lam P. (2013). The Need for International Terminology and Definitions for Texture-Modified Foods and Thickened Liquids Used in Dysphagia Management: Foundations of a Global Initiative. Curr. Phys. Med. Rehabil. Rep..

[31] Cichero J.A., Lam P., Steele C.M., Hanson B., Chen J., Dantas R.O., Duivestein J., Kayashita J., Lecko C., Murray J. (2017). Development of International Terminology and Definitions for Texture-Modified Foods and Thickened Fluids Used in Dysphagia Management: The IDDSI Framework. Dysphagia.

[32] Higashiguchi T., Ito A., Nishiyama H., Shigematsu T., Ishikawa A., Kato H., Iijima S., Kikuchi N. (2017). Appropriate nutritional management in patients with impaired mastication and those with mild dysphagia: A multicenter study of the usefulness of novel foods processed and softened by enzymes. Asia Pac. J. Clin. Nutr..

[33] Shimizu A., Momosaki R., Kayashita J., Fujishima I. (2020). Impact of Multiple Texture-Modified Diets on Oral Intake and Nutritional Status in Older Patients with Pneumonia: A Retrospective Cohort Study. Dysphagia.

